# STA-8666, a novel HSP90 inhibitor/SN-38 drug conjugate, causes complete tumor regression in preclinical mouse models of pediatric sarcoma

**DOI:** 10.18632/oncotarget.11869

**Published:** 2016-09-06

**Authors:** Christine M. Heske, Arnulfo Mendoza, Leah D. Edessa, Joshua T. Baumgart, Sunmin Lee, Jane Trepel, David A. Proia, Len Neckers, Lee J. Helman

**Affiliations:** ^1^ Pediatric Oncology Branch, National Cancer Institute, National Institutes of Health, Bethesda, MD, USA; ^2^ Developmental Therapeutics Branch, National Cancer Institute, National Institutes of Health, Bethesda, MD, USA; ^3^ Synta Pharmaceuticals, Lexington, MA, USA; ^4^ Urologic Oncology Branch, National Cancer Institute, National Institutes of Health, Bethesda, MD, USA

**Keywords:** sarcoma, SN-38, drug conjugate, topoisomerase 1 inhibitor, pediatric

## Abstract

Long-term survival in patients with metastatic, relapsed, or recurrent Ewing sarcoma and rhabdomyosarcoma is dismal. Irinotecan, a topoisomerase 1 inhibitor, has activity in these sarcomas, but due to poor bioavailability of its active metabolite (SN-38) has had limited clinical efficacy. In this study we have evaluated the efficacy and toxicity of STA-8666, a novel drug conjugate which uses an HSP90 inhibitor to facilitate intracellular, tumor-targeted delivery of the topoisomerase 1 inhibitor SN-38, thus preferentially delivering and concentrating SN-38 within tumor tissue. We present *in vivo* evidence from mouse xenograft models that STA-8666 results in more persistent inhibition of topoisomerase 1 and prolonged DNA damage compared to irinotecan. This translates into superior antitumor efficacy and survival in multiple aggressive models of both diseases in mouse xenografts, as well as in an irinotecan-resistant model of pediatric osteosarcoma, demonstrated by dramatic tumor shrinkage, durable remission and prolonged complete regressions following short-term treatment, compared to conventional irinotecan. Gene expression analysis performed on xenograft tumors treated with either irinotecan or STA-8666 showed that STA-8666 affected expression of DNA damage and repair genes more robustly than irinotecan. These results suggest that STA-8666 may be a promising new agent for patients with pediatric-type sarcoma.

## INTRODUCTION

Rhabdomyosarcoma (RMS) is the most common soft tissue sarcoma of childhood and Ewing sarcoma (ES) is the second most common malignant bone tumor of childhood [1, 2]. Outcomes in patients with these tumors who present with localized disease have improved over the past several decades, largely due to advances in multimodal therapy and supportive care [3]. However long-term outcomes in patients with metastatic, relapsed, or recurrent ES and RMS are dismal, with survival rates less than 20% [4]. Hence, new therapies for these diseases are critically needed.

Irinotecan, an analog of camptothecin, is an antineoplastic agent that acts to inhibit topoisomerase 1, resulting in impaired relaxation of supercoiled DNA during DNA replication [5]. It is commonly used as part of a chemotherapeutic regimen to treat adult patients with colorectal cancer, pancreatic cancer, small cell lung cancer and gastric cancer [6–9]. Likewise, irinotecan has shown promising activity in pediatric patients with relapsed and refractory sarcomas. Response rates of 11–86% for rhabdomyosarcoma [10–12] and 38% in Ewing/PNET [11] have been reported in heavily pretreated patients who received single-agent irinotecan on a variety of dosing schedules. When used in combination with other agents in early phase studies of relapsed Ewing sarcoma, response rates approached 60–70% in some studies [13]. Of note, studies in xenograft models of human tumors have shown that the activity of irinotecan is schedule-dependent, with protracted schedules producing superior responses [14]. Based on such data, protracted dosing schedules are typically used in pediatrics to maximize irinotecan exposure and efficacy [11, 15–17].

Despite the promising results seen in the clinical setting, there are a number of factors that negatively impact the efficacy of irinotecan, limiting its therapeutic potential. Irinotecan is a prodrug of SN-38 [18]. Compared to irinotecan, SN-38 has between a 100 and 1000-fold greater antitumor activity *in vitro,* but cannot be administered directly to patients due to difficulties with solubility and toxicity [19, 20]. Specifically, SN-38 is unstable at physiological pH [20]. In order to be converted to SN-38, irinotecan requires de-esterification, primarily in the liver [18]. Unfortunately, this process is inefficient and only a small amount is converted to the active metabolite [20, 21]. In addition, there is wide interpatient variability in efficiency of conversion to the active metabolite [20, 22]. Furthermore, irinotecan is also converted into a number of less active forms, including SN-38G, which is excreted in the bile and urine [21]. Due to the complex metabolism of irinotecan, drug bioavailability is not optimal in human patients.

Heat shock protein 90 (HSP90) is a molecular chaperone that regulates the post-translational folding, stability, and function of many client proteins. A number of these client proteins play critical roles in cancer cells, where HSP90 is widely expressed [23]. For this reason, HSP90 inhibitors have become an exciting target in cancer research. *In vivo*, it has been observed that HSP90 inhibitors negatively affect the growth of tumor cells, while minimally affecting normal tissues [24]. In fact, HSP90 inhibitors display very favorable pharmacokinetics for anticancer use, as they concentrate in tumor tissues for a prolonged period of time and at higher levels, compared to normal tissues [25–27]. Although the mechanism for this effect is not completely understood, it is characteristic of a number of chemically distinct clinically evaluated agents. Various theories for the tumor selectivity of HSP90 inhibitors include HSP90 overexpression, unique posttranslational modification, and high expression of Mps1 in tumor cells [28, 29]. Whatever its basis, this unique property makes HSP90 inhibitors ideal intracellular delivery vehicles for chemotherapeutic drugs, allowing for high tumor exposure and low systemic toxicity. STA-8666 (Synta Pharmaceuticals, Lexington, MA) is an HSP90 inhibitor drug conjugate (HDC), which consists of an HSP90 inhibitor (STA-8663) attached to SN-38 through a cleavable chemical linker. This agent has recently been shown to demonstrate prolonged tumor exposure to SN-38 when compared to irinotecan in several xenograft models [30, 31]. The purpose of this study was to test the efficacy of this HDC in xenograft models of irinotecan-responsive pediatric-type sarcomas, and to explore its mechanism of action in these tumor types.

## RESULTS

### The HSP90 inhibitor component of STA-8666 is less potent *in vitro* than the closely related HSP90 inhibitor in clinical use (ganetespib)

Published literature suggests that the HSP90 inhibitor component of STA-8666 functions primarily as a delivery vehicle [30, 31]. To investigate this presumptive mechanism of action in our pediatric-type sarcoma models, *in vitro* experiments were conducted comparing the activity of the HSP90 inhibitor fragment of STA-8666 (denoted STA-8663) to that of ganetespib (STA-9090), an HSP90 inhibitor currently undergoing clinical evaluation that has potent *in vitro* activity in pediatric sarcoma cell lines including TC32 (a Ewing sarcoma cell line) and RH30 (a translocation positive rhabdomyosarcoma cell line) (Supplementary Figure S1A and S1B). When assessed for effects on cell proliferation, STA-8663 was approximately 20-fold less potent than ganetespib in TC32 cells, and approximately 50-fold less potent in RH30 cells, suggesting that HSP90 inhibition *per se* likely plays a limited role in the anti-tumor activity of STA-8666.

Further analysis of the proposed mechanism was performed by assessing HSP70 induction, an accepted consequence and pharmacodynamic marker of robust HSP90 inhibition [32], in ES and RMS cells treated with ganetespib or STA-8663 (Supplementary Figure S2). In both TC32 and RH30 cells, induction of HSP70 occurred at ganetespib concentrations 100-fold lower than those of STA-8663, consistent with the cell proliferation data above and supporting a model in which HSP90 modulation by the HSP90 targeting component of STA-8666 is significantly less than that of ganetespib.

### STA-8666 results in complete regression of palpable ES and RMS tumors in SCID mice

**Figure 1:**
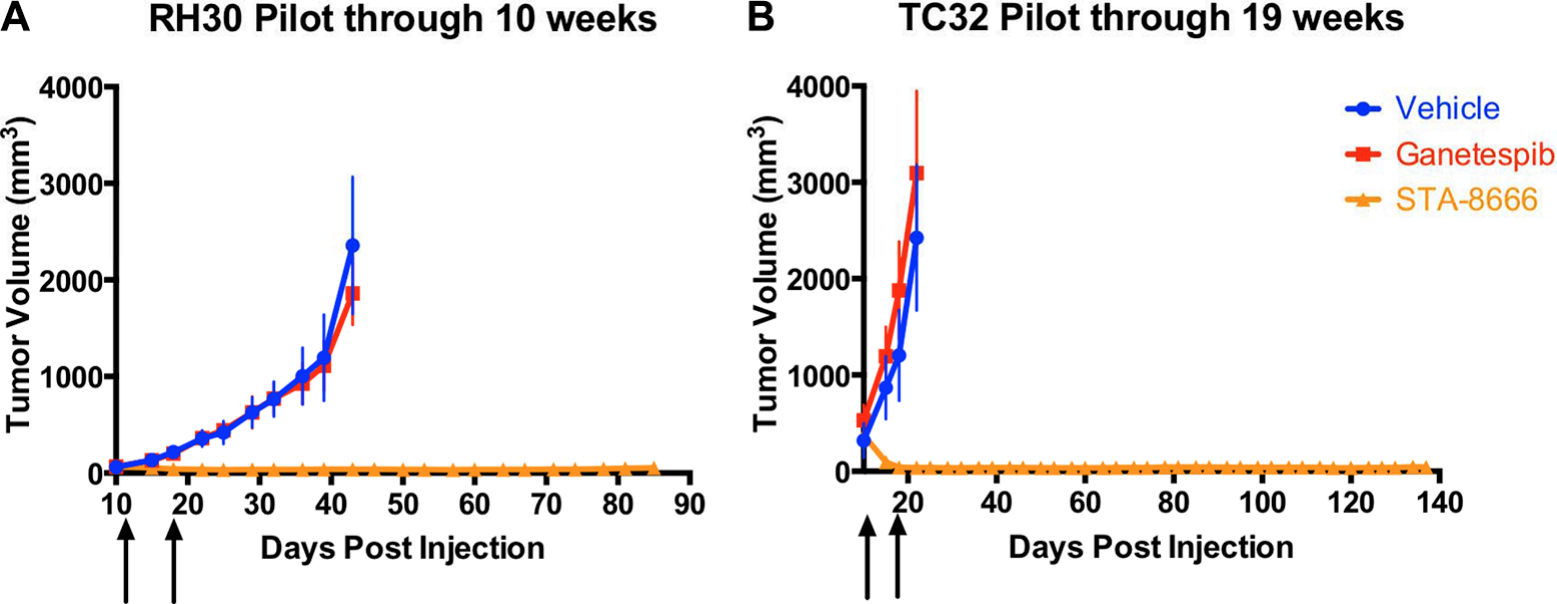
ES and RMS tumor volumes in pilot experiments with STA-8666 Tumor volumes of RH30 (RMS) (**A**) and TC32 (ES) (**B**) in mice treated with vehicle (blue), ganetespib at 50 mg/kg IV (red) or STA-8666 at 150 mg/kg IV (orange). Arrows indicate weekly treatments. Volume differences were statistically significant between the ganetespib and STA-8666 groups for days 15 to 43 in the RMS cohort (*p* ≤ 0.0002), and for days 15 to 22 in the ES cohort (*p* ≤ 0.0012).

An initial xenograft pilot experiment was conducted to compare the antitumor activity of STA-8666 to that of vehicle and an HSP90 inhibitor. STA-8663 was not available in quantities needed for xenograft experiments. Thus, we selected ganetespib for comparison. Based on the data described above, ganetespib is > 10-fold more potent than STA-8663, and would thus be expected to out-perform STA-8663 *in vivo* if HSP90 inhibition were a key contributor to efficacy in this setting. Treatment began 10 days after injection of cells, when tumors were palpable. Due to differences in growth rate of each tumor type, ES tumors were between 100 and 500 mm^3^ at the start of treatment and RMS tumors were between 50 and 90 mm^3^at the start of treatment. Of note, both cell lines were derived from patients who were irinotecan-naïve. Irinotecan was administered by the IP route, which has been shown to be more efficacious and less toxic than the PO and IV routes, respectively [21, 33]. Ganetespib and STA-8666 were administered by the IV route, following the recommendation of the manufacturer (Synta Pharmaceuticals). STA-8666 at a dose of 150 mg/kg (an equimolar dose of 75 mg/kg STA-8663), which is the maximally tolerated dose (MTD) for this agent in this strain of mouse, produced superior antitumor efficacy compared with vehicle and ganetespib. All mice in the STA-8666 group exhibited complete tumor regression after two doses, persisting for 78 days in all RMS mice and for 137 days in all ES mice. Following the same schedule, ganetespib at 50 mg/kg showed no antitumor activity in either xenograft model (Figure 1A and 1B). Unadjusted *p*-values were ≤ 0.0002 for comparisons made between tumor volumes of mice in the STA-8666 group and ganetespib group for days 15 to 43 in the RMS cohort, and ≤ 0.0012 for days 15 to 22 in the ES cohort.

### STA-8666 results in longer, dose-dependent remissions of large tumors and superior survival compared to irinotecan

**Figure 2:**
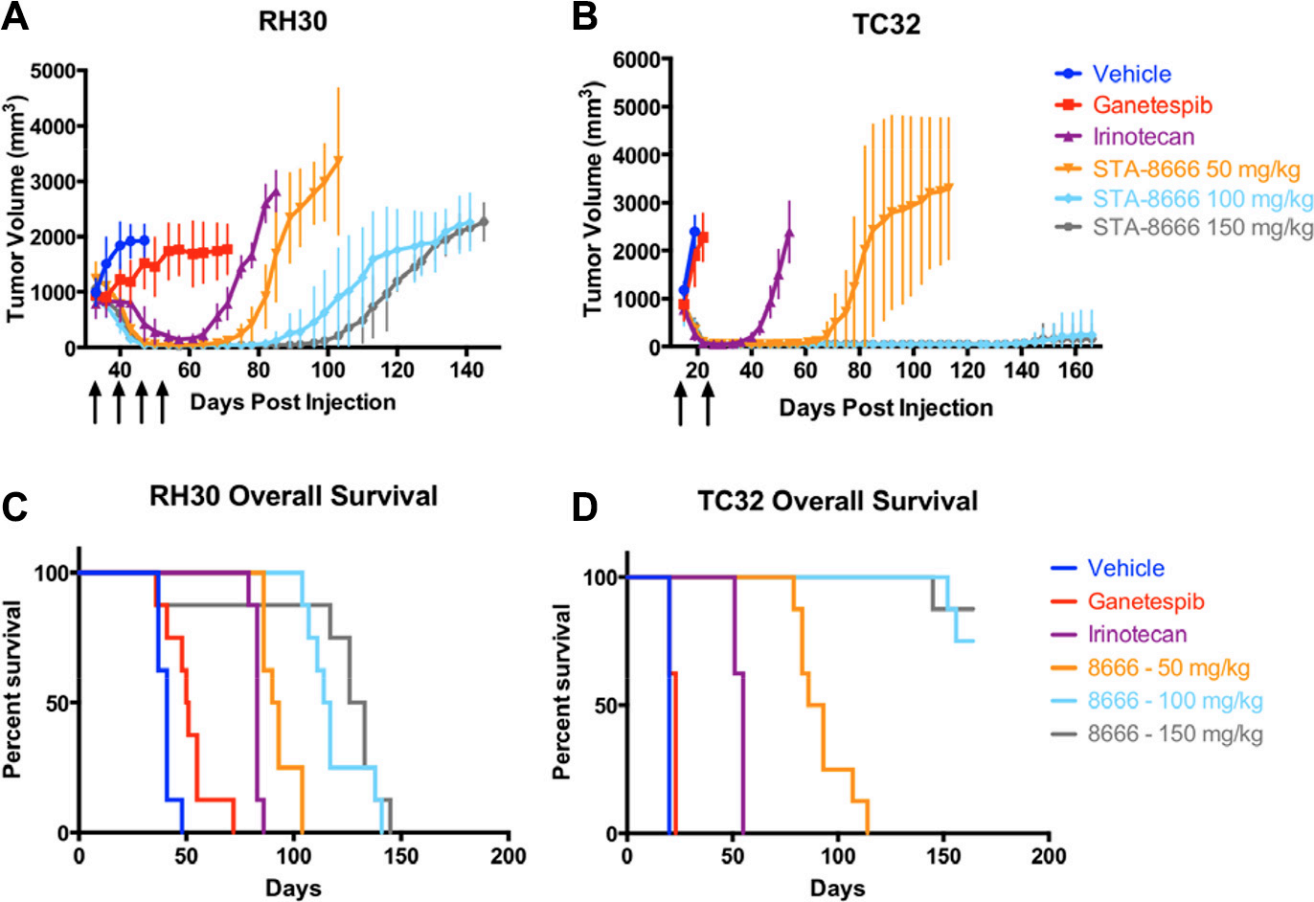
ES and RMS tumor volumes and Kaplan-Meier curves for mice treated with vehicle, ganetespib, high-dose irinotecan, or STA-8666 at varying doses Upper panel: Tumor volumes of RH30 (RMS) (**A**) and TC32 (ES) (**B**) in mice treated weekly with vehicle (dark blue), ganetespib at 150 mg/kg IV (red), irinotecan at 50 mg/kg IP (purple), STA-8666 at 50 mg/kg IV (orange), 100 mg/kg IV (light blue) and 150 mg/kg IV (gray). Arrows indicate weekly treatments for each experimental condition. Volume differences were statistically significant between the irinotecan group and the STA-8666 50 mg/kg group (*p* ≤ 0.007), the STA-8666 100 mg/kg group (*p* ≤ 0.0002), and the STA-8666 150 mg/kg group (*p* ≤ 0.0003) for days 64 through 85 in the RMS experiment. In the ES experiment, volume differences were statistically significant between the irinotecan group and the STA-8666 groups for all doses (*p* ≤ 0.0002) between days 36 and 54. Lower panel: Kaplan-Meier analysis of overall survival for mice with RH30 (**C**) and TC32 (**D**) tumors in the same experiment. Survival comparisons with irinotecan reached statistical significance in all groups (in RMS, *p* = 0.0004 for irinotecan v. STA-8666 at 50 mg/kg, *p* < 0.0001 for irinotecan v. STA-8666 at 100 mg/kg and irinotecan v. STA-8666 150 mg/kg; in ES, *p* < 0.0001 for irinotecan v. STA-8666 at all three doses). Dose responses on survival in both RMS (*p* = 0.0003) and ES (*p* < 0.0001) were statistically significant (Figure 2C and 2D).

Given the prolonged complete regressions of palpable tumors with STA-8666, we next determined whether postponing primary treatment until tumors were 1000 mm^3^ in ES and RMS xenografts would yield similar responses and whether a dose response would be observed. Additionally, a direct comparison of STA-8666 activity to high dose irinotecan and high dose ganetespib in both ES and RMS was undertaken. Tumors of both types were allowed to grow to between 800 and 1000 mm^3^ before treatment was begun. Mice were dosed weekly with vehicle, ganetespib, irinotecan, or STA-8666 at one of three doses. Dose selection was based upon the MTD for each agent in SCID-beige mice (150 mg/kg for ganetespib and STA-8666 and 50 mg/kg for irinotecan). In addition, STA-8666 was tested at two lower doses: 100 mg/kg, which is the equimolar dose of 50 mg/kg irinotecan and 50 mg/kg, which is half of the equimolar dose of irinotecan. The number of doses for each tumor type was determined by the response of the irinotecan arm; once the mice in the irinotecan arm no longer had palpable tumor, the dosing for all groups was discontinued. In both the irinotecan and STA-8666 arms, complete regressions were achieved in ES (after two doses) and RMS (after four doses). However, mice treated with STA-8666 at any dose had longer and more persistent remissions, compared to the irinotecan group (Figure 2A and 2B). Mice receiving irinotecan relapsed first (at day 61 in RMS and at day 29 in ES), while relapse in the lowest-dose (50 mg/kg) STA-8666 group was delayed (to day 68 in RMS and day 61 in ES). In RMS mice, a dose response effect was seen for STA-8666 with longest remissions noted in the 150 mg/kg group, followed by the 100 mg/kg group and then the 50 mg/kg group. In ES mice, 7/8 mice in both the 100 mg/kg and the 150 mg/kg groups had no tumor recurrence at 166 days. In the RMS experiment, unadjusted *p*-values were ≤ 0.007 for comparisons made between tumor volumes of mice in the irinotecan group and STA-8666 50 mg/kg group, ≤ 0.0002 for comparisons between the irinotecan group and the STA-8666 100 mg/kg group, and ≤ 0.0003 for comparisons between the irinotecan group and the STA-8666 150 mg/kg group for days 64 through 85. In the ES experiment, unadjusted *p*-values comparing tumor volumes of mice receiving irinotecan to those receiving STA-8666 were ≤ 0.0002 for all three dose comparisons between days 36 and 54. High-dose ganetespib (150 mg/kg) did not show significant antitumor activity in either group. Similar to the tumor growth rate, overall survival was significantly superior for the mice in the STA-8666 groups (in RMS, *p* = 0.0004 for irinotecan v. 50 mg/kg STA-8666, and *p* < 0.0001 for irinotecan v. STA-8666 at either 100 mg/kg or 150 mg/kg; in ES, *p* < 0.0001 for irinotecan v. STA-8666 at all three doses) with a dose response effect in both RMS (*p* = 0.0003) and ES (*p* < 0.0001) (Figure 2C and 2D).

### STA-8666 is superior to protracted irinotecan in an additional ES model

Understanding that a single weekly dose of irinotecan has less activity than protracted dosing in *in vivo* models [14], we next compared STA-8666 efficacy to protracted five day per week dosing of irinotecan to see if more frequent dosing of the topoisomerase inhibitor would minimize the difference in response between irinotecan and weekly STA-8666. In this experiment, EW8 (derived from an irinotecan-naïve patient) ES tumors were allowed to grow to an average of 600–800 mm^3^ before we began treatment with either vehicle, protracted dose irinotecan at 10 mg/kg daily, five days per week, or weekly STA-8666 at 150 mg/kg. MTD was again used to determine doses, and mice were dosed for two weeks.

**Figure 3:**
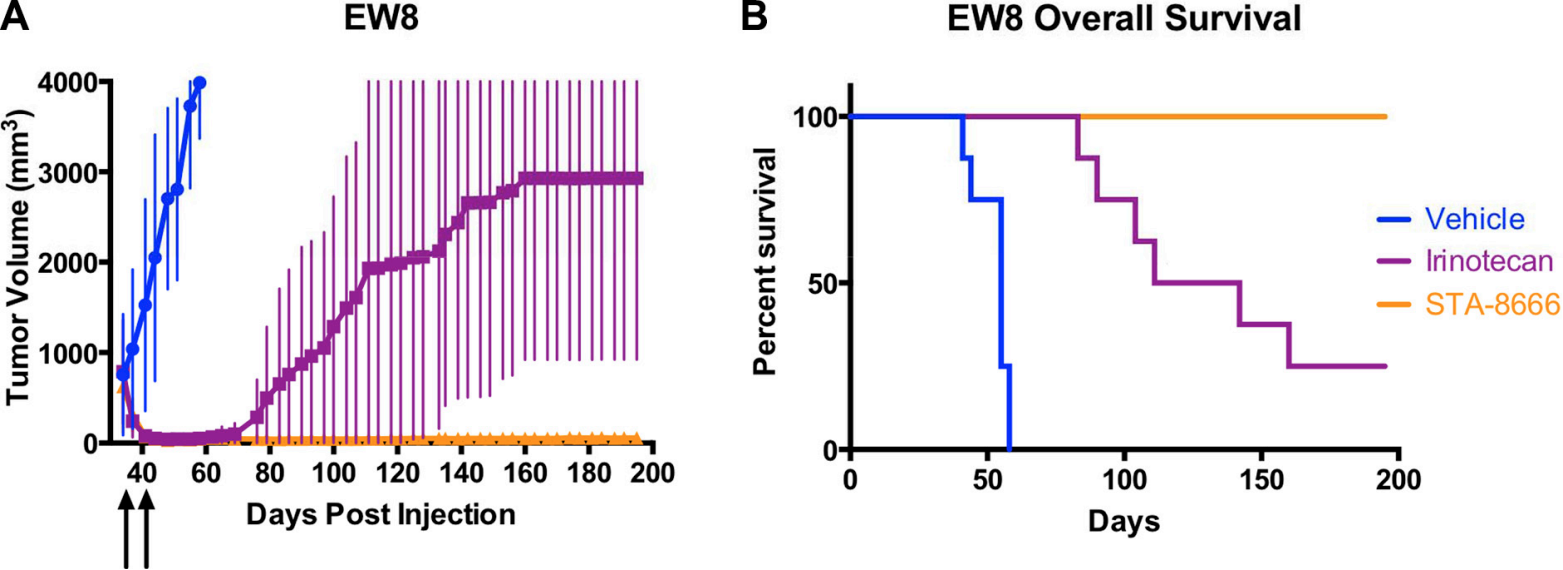
ES tumor volumes and Kaplan-Meier curves for mice treated with vehicle, protracted-dose irinotecan, or STA-8666 (**A**) Tumor volumes of EW8 xenografts in mice treated with vehicle (blue), irinotecan at 10 mg/kg IP daily for 5 days per week (purple), and STA-8666 at 150 mg/kg IV once per week (orange). The beginning of each treatment week is indicated by the arrows. Treatments in all groups lasted for two weeks. Volume differences were statistically significant between the irinotecan group and STA-8666 group (*p* ≤ 0.0379) for day 107 and beyond. (**B**) Kaplan-Meier analysis of overall survival for mice with EW8 tumors in the same experiment. Overall survival was significantly superior for the mice receiving STA-8666 (*p* = 0.0023).

As in the prior experiment, mice receiving both irinotecan and STA-8666 experienced rapid complete remissions within 10 days of beginning treatment (Figure 3A). Mice in the irinotecan group began relapsing at day 62 with 7/8 mice experiencing recurrence by day 181. In the STA-8666 group, all mice remained tumor-free at day 195. Unadjusted *p*-values were ≤ 0.0379 for comparisons made between tumor volumes of mice in the irinotecan group and STA-8666 group for day 107 and beyond. Overall survival was significantly superior for the mice receiving STA-8666 (*p* = 0.0023) (Figure 3B).

### STA-8666 is superior to protracted irinotecan and irinotecan plus ganetespib in PDX models of ES, but not in RMS

Given the striking results obtained with STA-8666 in the cell line-based xenograft models, we tested the agent in PDX models of both ES and RMS, as PDX models may be biologically different from xenografts derived from cell lines. Additionally, our PDX models were obtained from patients with highly aggressive metastatic disease. Of note, the RMS PDX was derived from a patient who had failed treatment with both irinotecan and a second camptothecin. The ES PDX was derived at diagnosis from a patient who was drug-naïve. For these experiments, we compared weekly STA-8666 to protracted dose irinotecan, and included an additional experimental arm in which mice received a combination of weekly ganetespib plus irinotecan on the protracted schedule to test whether there could be a combinatorial effect of the two drug classes comprising STA-8666. Tumors of both types grew to between 600 and 1000 mm^3^ before treatment began. Dosing was based on MTD values in the mice used for these experiments (NOG-F), which were lower than in the SCID-Beige strain. Mice received irinotecan at 7.5 mg/kg daily five days per week for two weeks, STA-8666 at 100 mg/kg once a week for two weeks, or the combination of ganetespib at 100 mg/kg once a week for two weeks plus the daily irinotecan.

**Figure 4:**
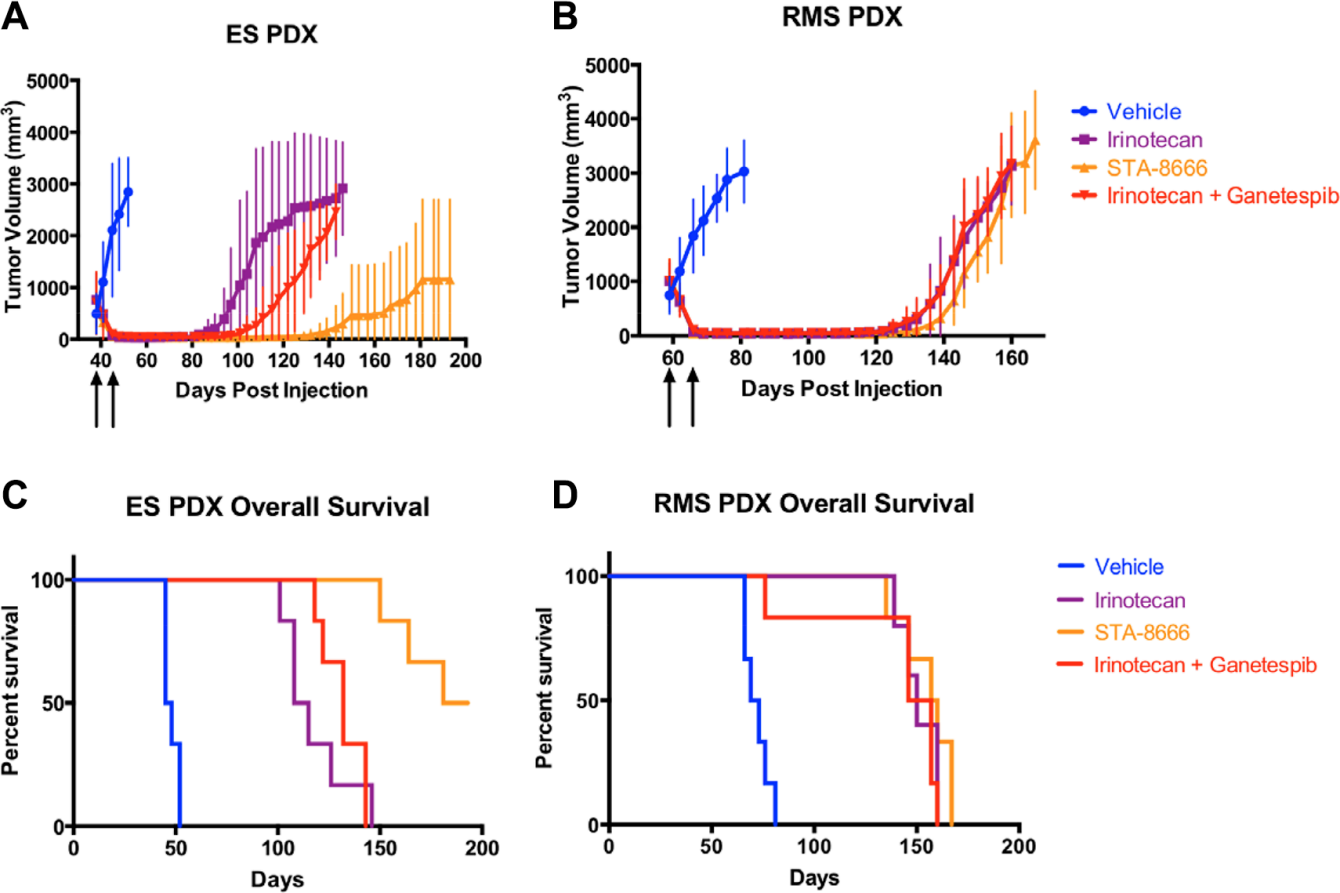
PDX tumor volumes and Kaplan-Meier curves for mice treated with vehicle, protracted-dose irinotecan, STA-8666, or ganetespib plus protracted-dose irinotecan Upper panel: Tumor volumes of ES PDX (**A**) and RMS PDX (**B**) in mice treated with vehicle (blue), irinotecan at 7.5 mg/kg IP daily for 5 days per week (purple), STA-8666 at 100 mg/kg IV once per week (orange), and irinotecan at the above schedule plus ganetespib at 100 mg/kg IV once per week (red). The beginning of each treatment week is indicated by the arrows. Treatments in all groups lasted for two weeks. For the EWS PDX, volume differences were statistically significant between the irinotecan group and STA-8666 group (*p* ≤ 0.0043) for days 87 through 146 and between the STA-8666 group and the combination group (*p* ≤ 0.0087) for days 94 through 143. *P*-values > 0.05 were obtained for comparisons of the irinotecan and combination groups during this same time period. For the RMS PDX, *p*-values > 0.05 were obtained for comparisons between each of the groups from days 118 through 167. Lower panel: Kaplan-Meier analysis of overall survival for mice with ES PDX (**C**) and RMS PDX (**D**) tumors in the same experiment. At end of experiment, the remainder of living mice had no evidence of tumor. Differences in overall survival between the STA-8666 group and other groups in ES were statistically significant (*p* = 0.0005 for irinotecan v. STA-8666; *p* = 0.0008 for STA-8666 v. combination), but no significance was seen for RMS (*p* = 0.3675 for irinotecan v. STA-8666; *p* = 0.1922 for STA-8666 v. combination).

Complete tumor regressions were achieved within two weeks in all mice in each of the three treatment arms. As in the prior experiment, mice with the ES PDX that received irinotecan relapsed first (beginning at day 81), mice that received the combination relapsed next (beginning at day 94), and mice that received STA-8666 relapsed last (beginning at day 111) or not at all. In the STA-8666 group, 3/6 mice had sustained complete remissions at day 195, when the experiment was terminated; this was not seen in either the irinotecan alone or combination group (Figure 4A). Unadjusted *p*-values were ≤ 0.0043 for comparisons made between tumor volumes of mice in the irinotecan group and STA-8666 group for days 87 through 146 and were ≤ 0.0087 for comparisons made between tumor volumes of mice in the STA-8666 group and the combination group for days 94 through 143. *P*-values > 0.05 were obtained for comparisons of the irinotecan and combination groups during this same time period. In contrast, mice harboring the RMS PDX and treated with irinotecan alone relapsed at the same time as those that received the combination (beginning at day 115). As in the ES PDX, mice that received STA-8666 relapsed last (day 126) (Figure 4B). *P*-values > 0.05 were obtained for comparisons between each of the groups from days 118 through 167. No RMS PDX mice in any of the treatment groups had sustained complete regressions. Finally, overall survival in the STA-8666 group was superior for ES (*p* = 0.0005 for irinotecan v. STA-8666; *p* = 0.0008 for STA-8666 v. combination), but not for RMS (*p* = 0.3675 for irinotecan v. STA-8666; *p* = 0.1922 for STA-8666 v. combination) (Figure 4C and 4D). These findings are consistent with our previous data suggesting that (1) ES tumors are more responsive to STA-8666 compared to RMS tumors, and (2) HSP90 inhibition *per se* contributed little to the anti-tumor activity of STA-8666 in either model.

### STA-8666 is effective in an irinotecan-resistant model of metastatic osteosarcoma

Because the RMS PDX was derived from a patient who had previously failed irinotecan, we investigated whether STA-8666 would be efficacious in an additional, non-RMS, irinotecan-resistant pediatric sarcoma model. For this experiment, we used HU09.H3, a highly aggressive cell line model of osteosarcoma (OS) that spontaneously metastasizes in mice. Although clinical data are limited, published data indicate that patients with OS generally respond poorly to irinotecan and irinotecan is not currently a component of OS treatment [12, 14, 34]. For this experiment, tumors were allowed to grow to an average of 750–900 mm^3^ and then mice began weekly treatment with vehicle, irinotecan, or STA-8666. MTD was used to determine doses as described earlier, and mice were dosed for two weeks.

**Figure 5:**
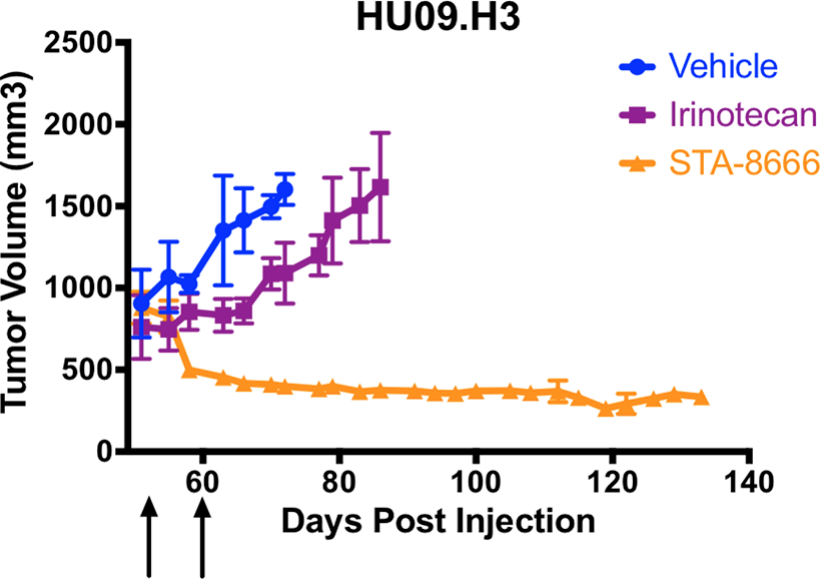
Osteosarcoma (OS) tumor volumes for mice treated with vehicle (blue), weekly irinotecan at 50 mg/kg IP (purple), and weekly STA-8666 at 150 mg/kg IV (orange) Arrows indicate weekly treatments for each experimental condition. Tumor volume differences approached but did not reach statistical significance (*p* = 0.0571), likely due to the smaller number of mice used in this experiment.

Unlike in prior experiments, mice in the irinotecan group did not experience tumor regression. Although irinotecan partially slowed tumor growth, irinotecan-treated mice survived only two weeks longer than mice in the vehicle group. However, mice treated with STA-8666 uniformly experienced durable tumor regression, with no evidence of regrowth at 134 days post-tumor cell inoculation, and superior survival (Figure 5). Due to the smaller number of mice in this experiment, statistical significance was approached (*p* = 0.0571) but not reached.

### STA-8666 is well tolerated in mice

Tolerability of STA-8666 was monitored by observation of overall health and weekly weights. Tolerability was excellent in all experiments performed with no changes in posture, coat or activity in any mice treated with the agent. In addition, there were no toxicity-related deaths or significant weight loss in treated mice at any of the dose levels tested at or below the established MTDs for each mouse strain (Supplementary Figure S3A and S3B).

### Duration of γH2AX expression suggests prolonged camptothecin effect in tumors treated with STA-8666

**Figure 6:**
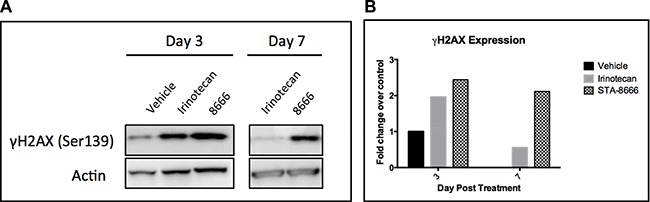
γH2AX expression in TC32 tumor samples at serial time points (**A**) Western blot showing that γH2AX is detectable longer in tumors from mice that received STA-8666, compared to irinotecan. Mice bearing TC32 tumors with an average size of 800 mm^3^ were treated with a single dose of either vehicle, irinotecan at 50 mg/kg IP or STA-8666 at 150 mg/kg IV. One mouse per group was sacrificed on day 3 and day 7. (**B**) Quantification of Western blot data normalized to actin. Signal in day 3 vehicle tumor was set to 1, as no vehicle mice remained alive on day 7.

The proposed mechanism of action of STA-8666 is based on enhanced delivery and sustained retention of SN-38 in tumor cells, compared to systemic administration of irinotecan. To explore the validity of this mechanism, we examined duration of expression of γH2AX, a pharmacodynamic marker of topoisomerase 1 inhibition [35], in tumor samples from treated mice. Mice bearing TC32 tumors of 800 mm^3^ were treated with a single dose of vehicle, irinotecan (IP at 50 mg/kg) or STA-8666 (IV at 150 mg/kg). One mouse per group was sacrificed on days 3 and 7. Tumor samples were processed for Western blot analysis of γH2AX expression (Figure 6). At day 3, γH2AX intensity in the tumor treated with STA-8666 was similar to that in the tumor treated with irinotecan. However, on day 7, while minimal γH2AX was detected in the irinotecan tumor, it was still readily detectable in the STA-8666 sample. This is consistent with published data in breast cancer xenografts showing that γH2AX peaked 1 day post irinotecan treatment and 7–10 days post STA-8666 treatment [30], and suggests that in this ES sarcoma model, STA-8666 likewise causes sustained inhibition of topoisomerase 1, compared with irinotecan.

### Gene expression analysis reveals overlapping profile for STA-8666 and irinotecan treated tumors

**Figure 7:**
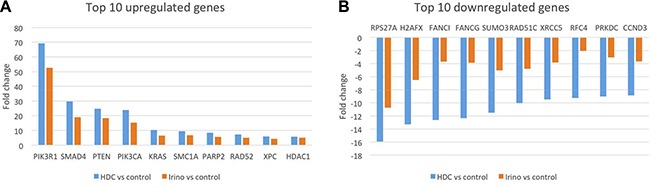
Fold-change comparison of gene expression in tumors five days after a single treatment with STA-8666 or irinotecan (**A**) Comparison of top 10 upregulated genes by fold-change versus vehicle in TC32 xenograft tumors treated with a single dose of either STA-8666 (100 mg/kg) or irinotecan (50 mg/kg) five days before tumor harvest. (**B**) Comparison of top 10 downregulated genes by fold-change versus vehicle in TC32 xenograft tumors treated with a single dose of either STA-8666 (100 mg/kg) or irinotecan (50 mg/kg) five days before tumor harvest.

As an additional method to investigate the mechanism of action of STA-8666, RNA expression analysis of a panel of 180 known DNA damage and repair genes was performed on TC32 xenograft tumors harvested five days after treatment with a single dose of vehicle, ganetespib (IV, 150 mg/kg), irinotecan (IP, 50 mg/kg) or STA-8666 (IV, 100 mg/kg). Of note, dose levels of irinotecan and STA-8666 in this experiment were equimolar. Analysis of target genes showed an overlapping profile between irinotecan and STA-8666 treated tumors, with the same top 10 upregulated and downregulated genes shared between the two groups (Figure 7A and 7B, and Supplementary Figure S4). For each of these 20 genes, but particularly for the 10 downregulated genes, the fold-change compared to vehicle control was greater for the STA-8666 treated tumors than for the irinotecan treated tumors, suggesting that, 5 days after treatment, STA-8666 affects expression of DNA damage and repair genes more robustly than does irinotecan administered at an equimolar dose. Further, the gene expression profile of tumors treated with ganetespib overlapped that of vehicle treated tumors, supporting the hypothesis that the HSP90 inhibitor component of STA-8666 does not contribute to the DNA damaging capacity of this agent (Supplementary Figure S4). A complete list of the genes showing differential expression compared to control, for tumors treated with irinotecan or STA-8666 can be found in Supplementary Figure S5.

## DISCUSSION

Irinotecan has emerged as a promising agent for patients with a variety of cancers that occur both in the adult population (e.g., colorectal, pancreatic, small cell lung and gastric cancers) and in pediatric patients (including sarcomas, neuroblastoma, hepatoblastoma and malignant glioma). However, challenges regarding the bioavailability of SN-38 have limited the potential of irinotecan in the clinic. Furthermore, when metabolized, irinotecan causes significant systemic toxicity, specifically myelosuppression and gastrointestinal toxicity (abdominal pain, nausea/vomiting, cholinergic syndrome, diarrhea) [10–12, 22, 34], thus reducing tolerability and limiting the possibility of dose escalation. More importantly, these toxicities overlap with the side effects of other cytotoxic therapies to negatively affect the optimal use of irinotecan in combination with other agents.

Over the last decade, investigators have attempted to find alternative methods to deliver irinotecan and SN-38 in ways that would maximize efficacy and minimize toxicity. Based on data from preclinical and clinical studies, ideal properties for delivery of SN-38 would include ready bioavailability, long exposure time for tumor tissue, and minimal effect on normal tissue [14, 21]. A number of new investigational approaches for SN-38 and irinotecan delivery have been developed which include both non-specific and tumor-specific prodrugs, nanomedicine formulations such as micelles, liposomes and nanoparticles, and drug-antibody-conjugates [20]. Preclinical data with nanomedicine formulations of irinotecan have shown improved retention in the circulation compared to conventional irinotecan. However, these agents are still challenged by the other limitations of irinotecan bioavailability, as the active agent is still irinotecan, and not the more highly potent SN-38 [19, 36–39]. Several of these agents are undergoing evaluation in clinical trials [40–42] and one (Onivyde), a liposomal form of irinotecan, was recently approved by the FDA. Antibody conjugates with SN-38, such as the anti-trop-2 ADC (sacituzumab govitecan), show encouraging activity in early phase clinical trials, but these agents rely on antibody targeting of surface proteins common to specific tumor types [43, 44]. Given the heterogeneous group of irinotecan-responsive cancers, an antibody-based approach may not be the optimal delivery choice for either the adult or pediatric patient population.

In this study we have evaluated the efficacy and toxicity of STA-8666, a unique drug conjugate that relies on HSP90-based intracellular delivery and prolonged tumor retention of SN-38, in multiple mouse xenograft models of pediatric-type sarcoma. In the majority of the models tested, all dose levels of STA-8666 resulted in superior tumor regression, longer time to relapse, and markedly prolonged overall survival compared with the HSP90 inhibitor ganetespib, irinotecan administered on a weekly schedule, irinotecan administered on a protracted schedule, and irinotecan administered in combination with ganetespib. In addition, STA-8666 treatment resulted in sustained complete regressions after minimal dosing in 75% of mice with ES (34/45 mice across all dose levels and models), and in 5% of mice with RMS (2/37 mice across all dose levels and models), whereas among the 47 mice treated with irinotecan, there was only one sustained complete regression observed (in an EW8-bearing mouse treated with protracted irinotecan). Further, treatment with STA-8666 in an irinotecan-resistant OS model surprisingly resulted in sustained complete regressions in all mice.

Several dosing regimens were tested in an effort to thoroughly compare the activity of STA-8666 to irinotecan, the clinically available form of SN-38. When equimolar doses (100 mg/kg STA-8666 and 50 mg/kg irinotecan) were given, STA-8666 was more efficacious in both ES and RMS, resulting in later relapse and prolonged overall survival. Furthermore, STA-8666 retained superior efficacy in both ES and RMS, even when tested at half the equimolar dose of irinotecan. However, since the presumed advantage of STA-8666 is related to tumor localization and duration of exposure, using equimolar doses may not be the most appropriate way to compare these agents. Thus, we additionally compared a protracted dosing schedule of irinotecan to weekly STA-8666 using the MTDs of each agent in the specific strains of mice, which is a clinically relevant means for comparison. In all but one case (RMS PDX, derived from an irinotecan-refractory tumor), STA-8666 resulted in superior outcomes, compared to irinotecan.

Data describing the pharmacokinetic/pharmacodynamic relationship of STA-8666 in breast cancer models indicate that SN-38 from STA-8666 is detectable at high nanomolar levels in tumors 10 days post-dose, which correlates with the continued presence of high nuclear γH2AX expression. In contrast, SN-38 is undetectable in tumors 3 days post-irinotecan, consistent with the rapid drop in nuclear γH2AX expression [30]. Although tumor pharmacokinetics were not directly studied in our sarcoma models, pharmacodynamic analysis of γH2AX expression in ES xenograft tumors supports this mechanism. Specifically, γH2AX was readily detected in tumor samples from mice 7 days after a single dose of STA-8666, whereas mice receiving irinotecan had no detectable γH2AX at this time point. Analysis of γH2AX expression in our models at later time points was limited by the sensitivity of our models to STA-8666, since the resultant rapid tumor shrinkage impeded tissue collection beyond 7 days post-treatment. The observation that none of the STA-8666 treated mice experienced clinical signs of toxicity during or after treatment suggests that even in the context of prolonged topoisomerase 1 inhibition in the tumor, STA-8666 may spare normal tissue from such exposure. STA-8666 thus meets the aforementioned criteria of an ideal SN-38 delivery system in that it is easily bioavailable, produces prolonged exposure in tumors and is well tolerated.

Results from the analysis of changes in RNA expression across a panel of known DNA damage and repair genes in tumors treated with STA-8666, irinotecan or ganetespib lend further support to the proposed mechanism of action. Further mechanistic insight may be gained from an examination of the functions of the most strongly affected genes, *PIK3R1* (expression increased 70-fold with STA-8666 and 50-fold with irinotecan) and *RPS27A* (expression decreased 16-fold with STA-8666 and 10-fold with irinotecan). While the expression of these genes has not been examined in ES, differential expression of both have been linked to certain cancers. *PIK3R1* encodes the regulatory subunit of PI3K (p85α), and reports in the literature have identified it as a tumor suppressor in a number of different malignancies, with low expression correlating with poor prognosis in breast cancer and renal cell carcinoma [45–47]. In contrast, *RPS27A* encodes a ribosomal protein that has been implicated in the promotion of proliferation and inhibition of apoptosis, and is upregulated in leukemia and colorectal cancer [48, 49]. Further investigation of the impact of topoisomerase 1 inhibition on these and other targets identified by this analysis is likely a fruitful avenue for future research.

Although further investigation in additional cell lines is necessary for confirmation, we noted a distinct difference between ES and RMS xenografts with respect to both rapidity and duration of response, with ES models (two cell line-derived xenografts and one PDX xenograft) being more responsive to STA-8666. This is likely partially related to differences in inherent sensitivity of each tumor type to SN-38, but other factors may be involved (e.g., prior treatment history in the case of the RMS PDX). Importantly, a third pediatric-type sarcoma model, osteosarcoma, although not particularly sensitive to systemic irinotecan, displayed remarkable *in vivo* sensitivity to STA-8666. In conclusion, we believe that the data presented here warrant clinical evaluation of STA-8666 in patients with pediatric-type sarcomas.

## MATERIALS AND METHODS

### Cell lines

RMS cell line RH30 has been previously described [50]. ES cell lines TC32 and EW8 have been previously described [51]. OS cell line HU09.H3 has been previously described [52]. The cells were maintained in RPMI growth medium (Life Technologies, Grand Island, NY) with 10% FBS, heat-inactivated (Sigma-Aldrich, St Louis, MO), 100 U/ml penicillin and 100 μg/ml streptomycin (Life Technologies), and 2 mM L-glutamine (Life Technologies) at 37°C in an atmosphere of 5% CO, prior to injection.

### Patient derived xenografts

Transplantable xenografts of primary human RMS (XEN-RMS-041) and ES (XEN-EWS-021) were obtained from the Molecular Oncology Section of the Pediatric Oncology Branch (CCR /NCI/National Institutes of Health). The creation of these xenografts was approved by the Institutional Review Board of the National Institutes of Health, and patients gave informed consent for their creation. Confirmation of molecular signature (EWSR1-FLI1 translocation for ES PDX, and PAX3-FOXO1 translocation for RMS PDX) was performed by PCR prior to injection.

### Animals

Animal studies were performed in accordance with the guidelines of the National Institutes of Health Animal Care and Use Committee. For experiments with TC32, EW8 and RH30 cell lines, four- to six-week-old female Fox Chase SCID beige mice (CB17.B6-Prkdc^scid^ Lyst^bg^ /Crl) were purchased from Charles River Laboratories (Wilmington, MA). Two million cells were put into a solution of HBSS and Geltrex LDEV-free reduced growth factor basement membrane matrix, mixed at a 1:1 ratio (Life Technologies), and injected orthotopically into the gastrocnemius muscle in the left hind leg. Mice were randomized after tumors developed, but prior to the start of treatment. Treatment with agents began on day 10, in the initial pilot experiment, when tumor was palpable, and on day 19 (TC32), day 33 (RH30), or day 34 (EW8) in the expanded experiments, when tumors were an average of 800–1000 mm^3^. For experiments with HU09.H3, two million cells were put into a solution of HBSS and injected orthotopically into the gastrocnemius muscle in the left hind leg of SCID beige mice (described above). Treatment with agents began on day 51, when tumors were an average of 750–900 mm^3^.

For PDX experiments, four- to six-week-old female NOG-F Homozygous/Homozygous NOD.Cg-*Prkdc^scid^ Il2rg^tm1Sug^*/JicTac mice were purchased from Taconic Biosciences (Derwood, MD). PDX cells were thawed and put into a solution of HBSS and Geltrex LDEV-free reduced growth factor basement membrane matrix, mixed at a 1:1 ratio. 100 uL of this solution was injected orthotopically into the gastrocnemius muscle in the left hind leg. Mice were randomized after tumors developed, but prior to the start of treatment. Treatment with agents began on day 38 for the ES PDX and day 59 for the RMS PDX, when tumors were an average of 600–1000 mm^3^.

All mice were maintained in a pathogen-free environment. Tumors were measured twice per week with calipers, and mice were monitored by observation of overall health and weekly weights to determine drug tolerability. Tumor volume was calculated by the following formula: *V* (mm3) = (*D x d2*)/6 × 3.14, Where *D* is the longest tumor axis and *d* is the shortest tumor axis. Tumors were harvested at midpoints and at study endpoint for biology studies. Treatment with ganetespib at 50 mg/kg (pilot experiments), 150 mg/kg (experiments with irinotecan arms) or 100 mg/kg (PDX experiments) and STA-8666 (50, 100 or 150 mg/kg) was given by tail vein injection on a weekly basis. Treatment with irinotecan was given at 50 mg/kg by intraperitoneal injection on a weekly basis (TC32, RH30, HU09.H3) or 10–mg/kg intraperitoneal injection daily five days per week for two weeks (EW8) for the cell line experiments, and at 7.5 mg/kg by intraperitoneal injection daily five days per week for two weeks for the PDX experiments.

### Compounds

Ganetespib and STA-8666 were provided by Synta Pharmaceuticals. Of note, the HSP90 inhibitor component of STA-8666 comprises 50% of the mass of this agent. The chemical structure of STA-8666 has been previously published [30]. Stock solutions were prepared by reconstitution in DMSO, aliquoted, and stored at −20°C. For *in vivo* studies, immediately prior to injection, aliquots were thawed and diluted 1:10 with 20% Kolliphor RH 40/80% D5W (Sigma Aldrich, St. Louis, MO). For STA-8666, a volume of 2N NaOH, equivalent to 1.6% of the total solution volume, was then added to ensure solubility. Irinotecan hydrochloride (20 mg/mL) was obtained from the Veterinary Pharmacy at the NIH and diluted to desired concentration with sterile saline immediately prior to injection.

### Protein analysis

Tumor lysates were prepared by measuring 50 mg of frozen tumor sample and homogenizing in 1 mL T-PER Tissue Protein Extraction Reagent (Life Technologies) plus phosphatase and protease inhibitors (Life Technologies). Protein lysates (30–100 *u*g/lane), quantified by BCA protein assay (Life Technologies), were separated by 4–12% SDS–PAGE (Life Technologies) and transferred to nitrocellulose membranes (Amersham Pharmacia Biotech, Piscataway, NJ). Membranes were blocked with 5% nonfat dried milk in TBS (KPL, Gaithersburg, MD)-Tween20 (Sigma Aldrich) (20 mM Tris-HCI, pH 7.5; 8 g/l of sodium chloride; 0.1% Tween 20) and then incubated with primary antibodies against phosphorylated histone γH2AX (Cell Signaling) at 1:1000.

### Xenograft statistical analysis

Tumor volumes were compared between groups using a Wilcoxon rank-sum test at serial time points selected to be appropriate according to the data being presented in each plot. Measurements for mice that had already reached endpoint were carried forward until all mice in the group had reached endpoint, or the experiment was terminated.

Mantel-Cox analysis was performed to compare survival of mice in each of the irinotecan groups to each of the comparison groups (Figures 2C, 2D, 3B, 4C and 4D). A log-rank test for trend was performed to compare doses of STA-8666 (Figure 2C and 2D).

### Gene expression analysis

Tumor xenograft samples were fixed in formalin for 16 hours and stored in 70% ethanol at 4°C. Total RNA was isolated using the RNeasy FFPE kit (Qiagen, Valencia, CA). RNA samples were analyzed on the NanoString nCounter Analysis platform (NanoString Technologies, Seattle, WA) for digital quantitation of expression of genes associated with DNA damage and repair (nCounter Vantage DNA Damage and Repair Panel, with probes for 180 target genes and 12 housekeeping genes). Each reaction contained 150 ng of total RNA in 7 ml aliquots, plus target-specific oligonucleotide probe pairs, fluorescently-labeled specific reporter tags, and a biotinylated universal capture tag. Analysis and normalization of the raw data were conducted using nSolver Analysis Software v2.6 (NanoString Technologies). Raw counts were normalized to internal levels. A background count level was estimated using the average count of the 8 negative control probes in every reaction plus 2 SDs.

All normalized 180 target gene copy number results from the NanoString analysis were imported into the Partek Genomics Suite (version 6.6, Partek Inc., St. Louis, MO) for determination of differential gene expression profiles using analysis of variance (ANOVA) based on treatment. Three samples treated once with irinotecan, STA-8666, or ganetespib five days prior to tumor harvest were compared to three samples treated once with vehicle five days prior to tumor harvest. ANOVA results were imported into Ingenuity Pathway Analysis (IPA) software (Ingenuity^®^ Systems, Redwood City, CA, http://www.ingenuity.com).

## SUPPLEMENTARY MATERIALS FIGURES


