# Supplementary material for: STA-8666, a novel HSP90 inhibitor/SN-38 drug conjugate, causes complete tumor regression in preclinical mouse models of pediatric sarcoma
Source: Oncotarget. 2016 Sep 6;7(40):65540–52. doi: 10.18632/oncotarget.11869 (PMC5323173; doi:10.18632/oncotarget.11869)
Supplement: Supplementary file 1 [file oncotarget-07-65540-s001.pdf]

STA-8666, a novel HSP90 inhibitor/SN-38 drug conjugate, causes complete tumor regression in preclinical mouse models of pediatric sarcoma

Supplementary Materials

MATERIALS AND METHODS

Compounds

Ganetespib, STA-8666 and STA-8663 were provided by Synta Pharmaceuticals. Stock solutions were prepared by reconstitution in DMSO, aliquoted, and stored at −20°C.

Cell proliferation assays

Two independent methods for determining cell proliferation were performed. Cells were plated at a density of 2,000 cells per well in 96-well plates overnight and treated for 72 hours. Cell survival was determined using MTT assay with 3-(4,5-dimethylthiazolyl-2)-2, 5-diphenytetrazolium bromide (Promega, Madison, WI), performed according to the manufacturer's instructions. In addition, cell growth was monitored in real time using the IncuCyte System (Essen BioScience, Ann Arbor, MI).

Protein analysis

Cell lysates were prepared by plating 5 million cells in 10-cm plates and allowing them to grow for 24 hours. Thereafter, cells were treated for an additional 24 hours. Cells were harvested with 1X cell lysis buffer (Cell Signaling, Beverly, MA) plus phosphatase and protease inhibitors (Life Technologies). Protein lysates (30 *ug*/lane), quantified by BCA protein assay (Life Technologies), were separated by 4–12% SDS–PAGE (Life Technologies) and transferred to nitrocellulose membranes (Amersham Pharmacia Biotech, Piscataway, NJ). Membranes were blocked with 5% nonfat dried milk in TBS (KPL, Gaithersburg, MD)-Tween20 (Sigma Aldrich) (20 mM Tris-HCI, pH 7.5; 8 g/l of sodium chloride; 0.1% Tween 20) and then incubated with primary antibodies against HSP70/HSP72 (Enzo Life Sciences, Farmingdale, NY) at 1:1000.

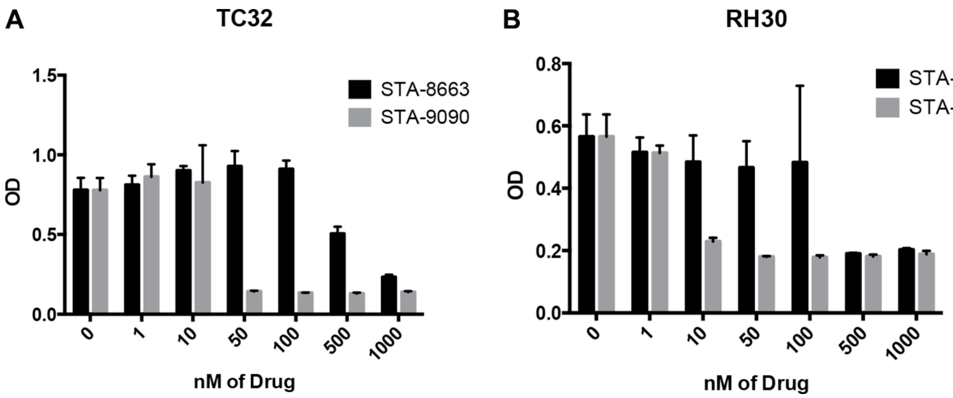

Supplementary Figure S1: Cell viability by MTT assay after treatment with STA-9090 or STA-8663 (the HSP90 inhibitor fragment of STA-8666) in TC32 ES cells (A) and RH30 RMS cells (B) at 72 hours. Cells were plated at a density of 2,000 cells per well in a 96-well plate and grown overnight. At 72 hours, cells were treated with varying concentrations of either STA-8663 or STA-9090 (ganetespib) for 72 hours. In TC32 cells, STA-8663 is 20 times less potent than STA-9090. In RH30 cells, STA-8663 is 50 times less potent than STA-9090.

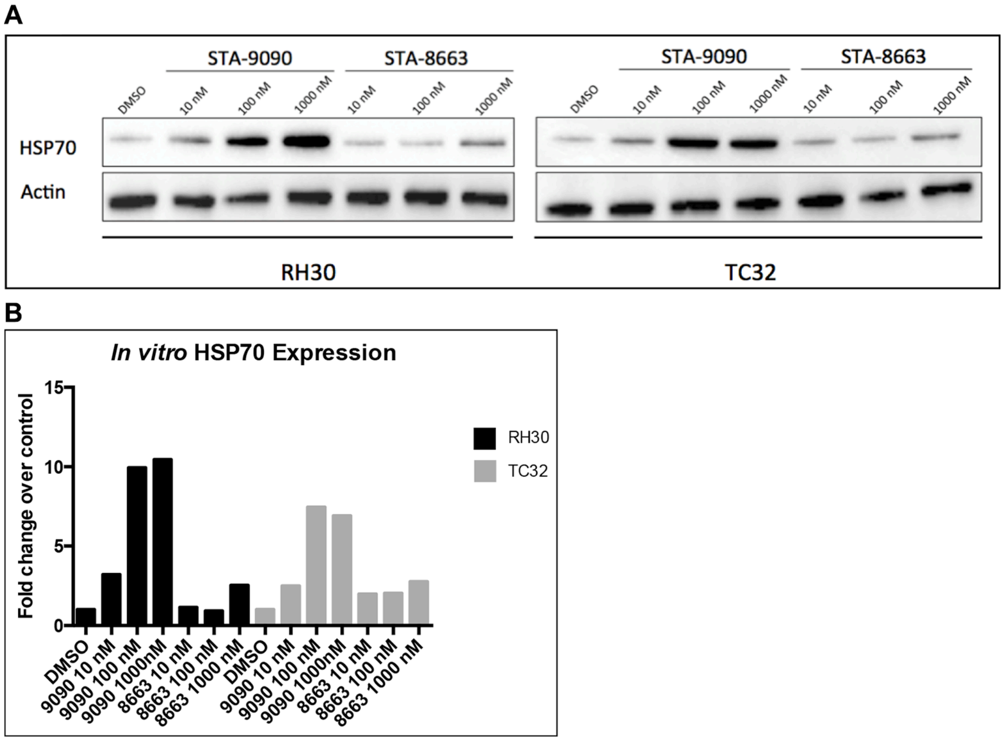

Supplementary Figure S2: (A) Western blots showing HSP70 activity after treatment with STA-9090 or STA-8663 (the HSP90 inhibitor fragment of STA-8666). Cells were treated for 24 hours with specified drug prior to lysis. In both RH30 RMS cells and TC32 ES cells, HSP70 induction occurs at doses 100-fold lower in STA-9090. (**B**) Quantification of Western blot.

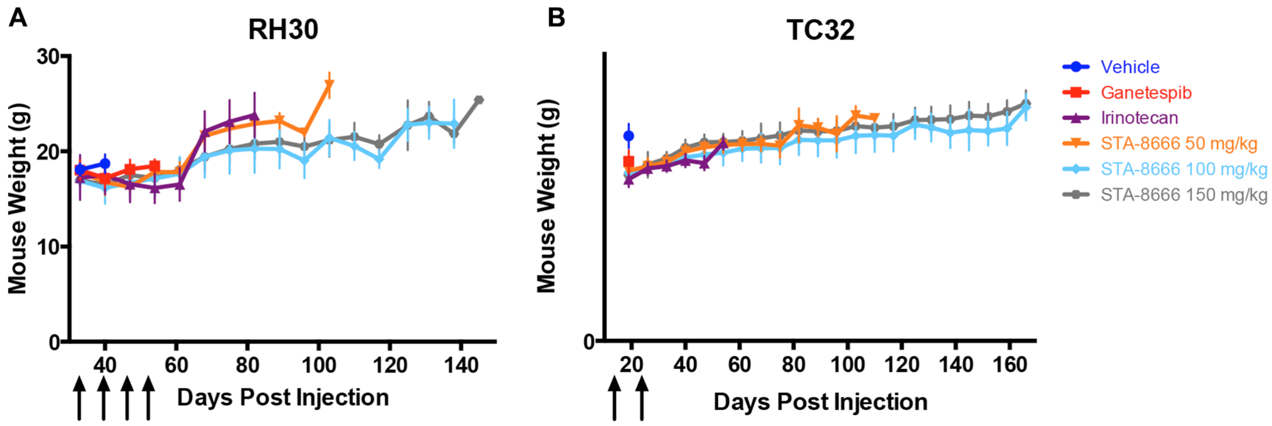

Supplementary Figure S3: Average mouse weights for experimental results shown in Figure 4. (**A**) RH30 bearing mice and (**B**) TC32 bearing mice treated weekly with vehicle (dark blue), ganetespib at 50 mg/kg IV (red), irinotecan at 50 mg/kg IP (purple), STA-8666 at 50 mg/kg IV (orange), 100 mg/kg IV (light blue) and 150 mg/kg IV (gray). Arrows indicate weekly treatments for each experimental condition.

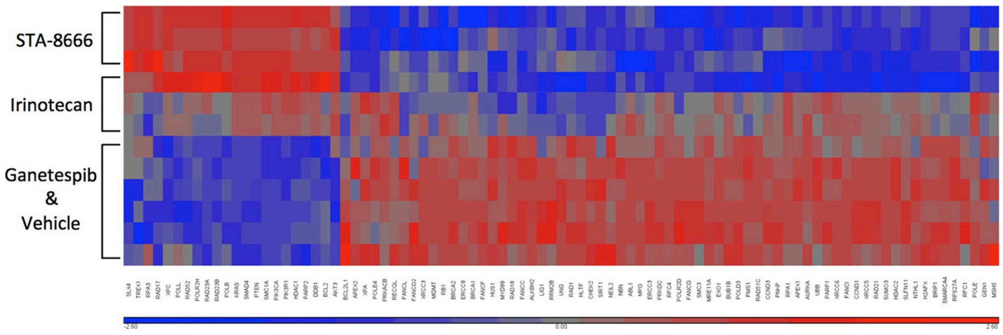

Supplementary Figure S4: Gene expression profile (using the NanoString DNA damage/response gene panel) of TC32 xenograft tumors treated with a single dose of either STA-8666 (100 mg/kg), irinotecan (50 mg/kg), ganetespib (150 mg/kg) or vehicle five days before tumor harvest.

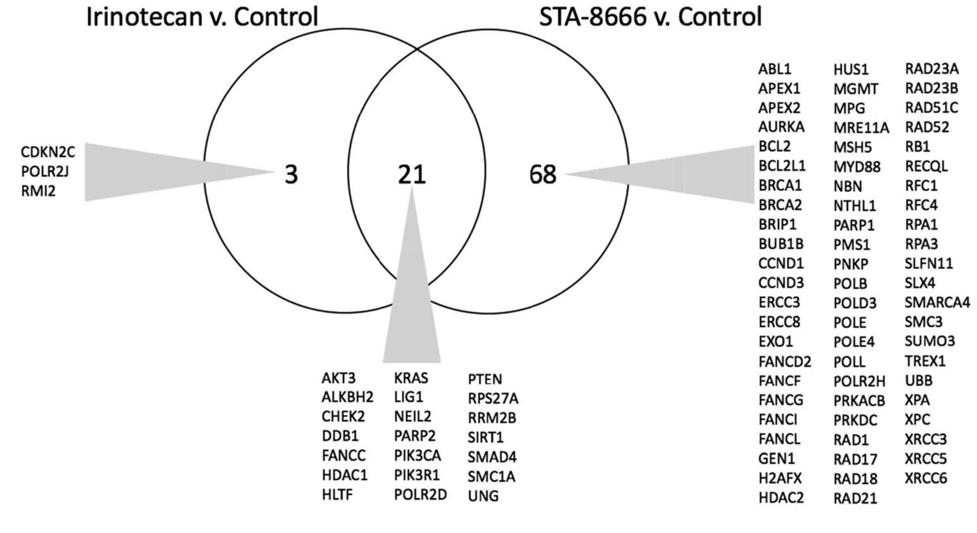

Supplementary Figure S5: Lists of differentially expressed DNA damage/repair related genes in TC32 tumors treated with irinotecan or STA-8666, when compared to vehicle controls. Of note, no differential effects were observed when comparing ganetespib treated tumors with vehicle controls (not shown).
